# Benefits of Structured and Free Monoacylglycerols to Deliver Eicosapentaenoic (EPA) in a Model of Lipid Malabsorption

**DOI:** 10.3390/nu4111781

**Published:** 2012-11-21

**Authors:** Cristina Cruz-Hernandez, Sagar K. Thakkar, Julie Moulin, Manuel Oliveira, Isabelle Masserey-Elmelegy, Fabiola Dionisi, Frédéric Destaillats

**Affiliations:** Nestlé Research Center, Vers-chez-les-Blanc, PO Box 44, CH-1000 Lausanne 26, Switzerland; Email: sagar.thakkar@rdls.nestle.com (S.K.T.); julie.moulin@rdls.nestle.com (J.M.); manuel.oliveira@rdls.nestle.com (M.O.); isabelle.masserey-elmelegy@rdls.nestle.com (I.M.-E.); fabiola.dionisi@rdls.nestle.com (F.D.); frederic.destaillats@rdls.nestle.com (F.D.)

**Keywords:** malabsorption, monoacylglycerol, long-chain polyunsaturated fatty acids, eicosapentaenoic acid, docosahexaenoic acid

## Abstract

In the present study, we used a preclinical model of induced lipolytic enzyme insufficiency, and hypothesized that the use of monoacylglycerols (MAG) will enhance their bioavailability and delivery to the tissues. Experimental diets containing 20% lipids were fed to rats for 21 days with or without Orlistat. The control diet of fish oil (FO), a source of EPA and DHA, was tested against: structured (**A**) vanillin acetal of *sn*-*2* MAG (Vanil + O) and (**B**) diacetyl derivative of *sn*-2 MAG (Acetyl + O) and (**C**) free MAG (MAG + O). FA profiles with an emphasis on EPA and DHA levels were determined in plasma, red blood cells (RBC), liver, spleen, brain and retina. We observed significant reduction of lipid absorption when rats co-consumed Orlistat. As expected, the FO groups with and without Orlistat showed the biggest difference. The Vanil + O, Acetyl + O and MAG + O groups, demonstrated higher levels of EPA (5.5 ± 1.9, 4.6 ± 1.6 and 5.6 ± 0.6, respectively) in RBC compared with FO + O diets (3.3 ± 0.2, 2.6 ± 0.2). Levels of EPA incorporation, in plasma, were similar to those obtained for RBC, and similar trends were observed for the collected tissues and even with DHA levels. These observations with two MAG derivatives providing the fatty acid esterified in the *sn*-2 position, show that these molecules are efficient vehicles of EPA in malabsorption conditions which is in line with our hypothesis. Free MAG, characterized as having exclusively *sn*-1(3) isomers of EPA, demonstrated better absorption efficiencies and accretion to tissues when compared to structured MAG. The study demonstrated that structured and free MAG can be used efficiently as an enteral vehicle to supply bioactive fatty acids such as EPA and DHA in lipid malabsorption where diminished lipolytic activity is the underlying cause.

## 1. Introduction

Defects in either intraluminal lipid digestion or uptake and transport of its digestive products across the gut barrier may lead to lipid malabsorption [1,2]. Clinically, lipid malabsorption is well recognized by steatorrhea and may bear severe consequences if the enzymatic activity in the upper intestine drops below 10% of what is considered normal [2,3]. Another example is cystic fibrosis, an autosomal recessive genetic disorder affecting multiple organ systems, most critically the lungs. However, it also exhibits pancreatic insufficiency that leads to malabsorption of dietary lipids. Enzyme replacement therapy though helpful still shows a certain degree of steatorrhea which indicates additional underlying causes of malabsorption. Indeed, it has been suggested that this type of persistent malabsorption is not only due to insufficient enzymes but also due to incomplete intraluminal solubilization or reduced enterocyte uptake, or both [2]. Another clinical scenario of lipid malabsorption is observed after intestinal surgeries. Gastrojejunostomy, ileostomy and similar surgeries may lead to reduced surface area for absorption, thereby creating a malabsorption condition. Likewise, cholecystectomy may also have an impact due to an impaired secretion of the bile required for emulsification of lipids for efficient lipolytic activity. In these and other malabsorption conditions, management is imperative as lipids are not only a source of energy, but also of bioactive nutrients. Minor lipids found in the human diet, for example, α-linolenic acid (ALA, 18:3 *n*-3) and linoleic acid (LA, 18:2 *n*-6) are truly essential because humans lack the enzymes required for their biosynthesis. These FA also are precursors to other biologically important LC-PUFA such as arachidonic acid (ARA, 20:4 *n*-6), eicosapentaenoic acid (EPA, 20:5 *n*-3) and docosahexaenoic acid (DHA, 22:6 *n*-3) that are proposed to have multiple benefits.

The bulk of dietary lipid is contributed by triacylglycerols (TAG). Distribution and composition of fatty acids (FA) in dietary TAG has an impact on their bioavailability and subsequently their exerted function on the body. During digestion, dietary TAG encounters the effects of gastrointestinal lipases resulting in formation of *sn*-2 monoacylglycerol (MAG) and two free FA that are absorbed by enterocytes [4]. Within enterocytes, the *sn*-2 MAG are reacylated to TAG and are released into lymphatic circulation via chylomicrons. FA released from the *sn*-1 and *sn*-3 positions often have different metabolic fates than FA at the *sn*-2 position as they are retained on the glycerol backbone in absence of *sn*-2 specific lipolytic enzymes. Therefore they cross apical barrier as 2-MAG, exerting different solubility than free FA [5]. The metabolic fates and hydrolysis rates depend on the FA chain length, nature of the FA in the TAG molecule and stereospecific (*sn*) location on the TAG [4,6]. Indeed studies have shown that the use of interesterified lipids with modified FA composition (*i.e.*, palmitic acid, stearic acid) at the *sn*-2 position may affect the amount absorbed [1,7].

Researchers have employed different strategies of preclinical models to study lipid malabsorption. One employs partial small bowel resection in rats [8] to reduce absorptive surface area. Alternatively, if the interest is primarily in cystic fibrosis, a knockout mice model of cystic fibrosis transmembrane conductance regulator (CFTR) may be used [9]. Finally, a lipase inhibiting compound like Orlistat (40% lower lipid absorption) may be used to create lipid malabsorption like condition when dietary lipids are provided as TAG in rats [6,10]. It is imperative that the selection of the model should be commensurate with the hypothesis being tested.

Current strategies to combat lipid malabsorption may include dietary supplements, intestinal enzyme therapy and treating underlying causes such as inflammation. Alternatively, dietary solutions can be sought to enhance absorption in deficient conditions. In case of enzyme insufficiency, administration of partially digested TAG, such as MAG, may help intraluminal solubilization and enterocyte uptake. Therefore, the objective of the present study was to identify if MAG provided in different forms are potential vehicles of fatty acids in conditions of low lipases activity.

**Figure 1:**
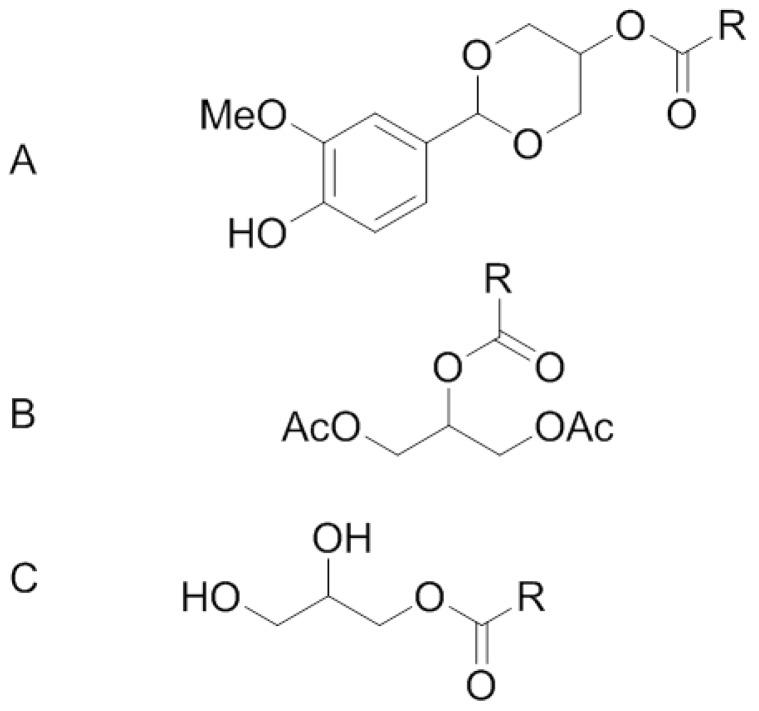
Chemical structures and the name of the compounds tested in the present study: (**A**) Vanillin acetal of *sn*-2-MAG; (**B**) 1,3-diacetyl-2-MAG; (**C**) *sn*-1(3)-MAG; R indicates a fatty acid as eicosapentaenoic (EPA) or docosahexaenoic acid (DHA).

Generally MAG oils can be produced by esterification of FA with glycerol followed by short-path distillation purification [11]. MAG can occur as two different type of positional isomers: *sn*-1(3)-MAG and *sn*-2-MAG. Unsaturated *sn*-2-MAG are not stable and readily isomerized to give rise to *sn*-1(3)-MAG [12]. Therefore, *sn*-2-MAG cannot be used in feeding experiment as free form and must be chemically protected in order to remain stable during the trial. In the present study, two protective agents have been used to stabilize *sn*-2 MAG: esterification with acetic acid which leads to formation of diacethyl-MAG derivatives and protection with vanillin giving rise to the formation of an acetal (Figure 1). In addition to these protected *sn*-2-MAG variants we also compared a free MAG by feeding rats receiving Orlistat. We assessed the accretion of the FA of interest, EPA in plasma and RBC for 21 days.

## 2. Methods and Materials

### 2.1. animals and Experimental Diet

All experimental procedures involving animals were approved by the cantonal veterinary office in Switzerland and revised by the internal ethical committee of the Nestlé Research Center. Male Wistar rats (*n* = 30, 270 ± 5 g) were housed in independent cages (Euro standard Type III H: 425 × 266 × 185 mm, floor area 800 cm^2^, Techniplast, Switzerland), received tap water and diets *ad libitum* for 21 days. The animals were randomly divided into five groups. 

Fish Oil (FO) diet consisted of AIN 93M base diet supplemented with fish oil (Sofinol S.A., Manno, Switzerland), high oleic sunflower oil (HOSO, Sofinol S.A., Manno, Switzerland) and cocoa butter (Gerkens Cacao^®^, Deventer, The Netherlands) as a lipid source. Total lipid content was 20 g/100 g diet (approximately 40% of the energy), see Table 1. Diets Fish oil + Orlistat (FO + O), free MAG + O, *sn*-2 MAG vanillin acetal + Orlistat (Vanil + O) and Acetylated *sn*-2 MAG + Orlistat (Acetyl + O) were AIN 93M base diet supplemented with FO, HOSO and either MAG Vanillin Acetal (Stepan Co., Northfield, IL, USA), Diacetylated MAG (Stepan Co., Northfield, IL, USA) or free MAG (Cognis GmbH, Illertissen, Germany) + Orlistat (MAG + O) containing 95.8% of α-MAG, as previously characterized [9] (Figure 1), plus 400 mg of Orlistat/kg of diet as listed in Table 1. The diets were prepared by mixing all the ingredients except Orlistat. The homogenized powders were dried at a low temperature and stored in a small sachet under vacuum at −20 °C. These special precautions were taken to avoid oxidative degradation of *n*-3 LC-PUFA. All animals were fed with a paste (water/powder diet, 1:1 w/w) prepared freshly every day. For the groups receiving Orlistat, the drug was added to the diet at 400 mg/kg every time the paste was prepared. FA composition of diets is described in Table 2.

**Table 1:** Composition of the experimental diets (g/kg of dry matter).

Nutrients (g/kg)	FO	FO + O	Vanil + O	Acetyl + O	MAG + O
Lipids (Total)	200	200	200	200	200
Cocoa butter	100	100	100	100	100
High Oleic Sunflower oil	72	72	60	70	79
Fish oil	28	28	-	-	-
MAG Vanillin Acetal	-	-	40	-	-
Diacetylated MAG	-	-	-	30	-
MAG	-	-	-	-	21
EPA (20:5 *n*-3)	4.4	4.4	4.4	4.2	4.7
DHA (22:6 *n*-3)	2.9	2.9	7.4	3.6	3.1
Orlistat	-	0. 4	0. 4	0. 4	0. 4
Corn starch	461	461	461	461	461
α-Casein	140	140	140	140	140
Sucrose	100	100	100	100	100
Cellulose	50	50	50	50	50
Mineral mix AIN-93M	35	35	35	35	35
Vitamin mix AIN-93M	10	10	10	10	10
l-cysteine	1.8	1.8	1.8	1.8	1.8
Choline bitartrate	2.5	2.5	2.5	2.5	2.5
Butylhydroxytoluene	0.008	0.008	0.008	0.008	0.008

(1) FO + O = Fish oil + Orlistat, Vanil + O = MAG Vanillin Acetal + Orlistat, Acetyl + O = Diacetylated MAG + Orlistat, MAG + O = MAG + Orlistat; (2) Cocoa butter was obtained from Gerkens Cacao (Deventer , The Netherlands) and HOSO and fish oil from Sofinol (Nestlé, Lausanne, Switzerland). Orlistat, gastric and pancreatic lipases inhibitor by Roche (Basel, Switzerland); (3) Vanillin acetal of 2-MAG (Stepan Co., Northfield, IL, USA), 1,3-diacetyl-2-MAG (Stepan Co., Northfield, IL, USA), MAG (Cognis GmbH, Illertissen, Germany).

**Table 2:** Weight Parameters, Dietary Intake and Apparent Lipid Absorption in rats fed different oils for 21 days.

		Experimental Groups				
		FO	FO + O	Vanil + O	Acetyl + O	MAG + O
		Mean ± SD	Mean ± SD	Mean ± SD	Mean ± SD	Mean ± SD
Final body weight (g)		317.7 ± 13.1	310.6 ± 7.6	314.6 ± 4.2	316.5 ± 8.8	310.4 ± 13.6
Weight gain (g/day)		4.4 ± 0.6	4.0 ± 0.3	4.2 ± 0.7	4.6 ± 0.3	4.1 ± 0.5
Fat Mass (g)	Day 0	30.7 ± 1.6	30.1 ± 0.4	29.0 ± 1.8	29.1 ± 0.7	29.4 ± 0.8
	Day 21	54.4 ± 4.0	46.1 ± 1.3	49.6 ± 2.7	52.3 ± 3.0	50.2 ± 5.6
Lean Mass (g)	Day 0	179.0 ± 5.6	181.2 ± 3.7	181.6 ± 3.5	178.6 ± 4.6	178.6 ± 4.7
	Day 21	223.8 ± 8.8	225.2 ± 5.8	226.3 ± 3.1	224.8 ± 4.9	220.1 ± 7.7
Lipid Intake (g/day)		4.1 ± 0.2	4.6 ± 0.2	5.2 ± 0.1 *	4.9 ± 0.3	4.9 ± 0.3
Daily Food Intake (g/day)		20.7 ± 0.8	22.8 ± 0.9	25.0 ± 0.6 *	23.9 ± 1.4	23.9 ± 1.7
Fecal Lipid excretion (g/day)		0.43 ± 0.05 ***	2.49 ± 0.30	3.10 ± 0.17 ***	1.97 ± 0.21 ***	3.16 ± 0.31 ***
Apparent Lipid absorption (%)		89.5 ± 0.8 ***	44.8 ± 4.7	37.1 ± 2.1 ***	58.5 ± 2.5 ***	32.9 ± 3.6 ***
EPA Intake (mg/day)		91.08 ± 3.5	100.32 ± 4.2	110.0 ± 3.2	100.38 ± 6.2	112.33 ± 7.5
DHA Intake (mg/day)		60.03 ± 3.5	66.12 ± 4.2	185 ± 3.2 ***	86.04 ± 6.2 **	74.09 ± 5.3

*, **, *** indicate *P* < 0.05, 0.01 and 0.001, respectively; FO + O = Fish oil + Orlistat, Vanil + O = MAG Vanillin Acetal + Orlistat, Acetyl + O = Diacetylated MAG + Orlistat, MAG + O = MAG + Orlistat; Apparent fat absorption = 100 × [(fat intake − fecal fat excretion)/fat intake]; Values are means ± S.E.M. (*n* = 6). For statistics, data for groups fed fish oil diet or diets supplemented with MAG and MAG derivatives + Orlistat have been compared to the group receiving fish oil + Orlistat.

### 2.2. Experimental Design

Body weight was recorded twice a week for 21 days. Food intake was recorded five times a week. Body composition (fat and lean mass) was determined in animals in triplicate by quantitative nuclear magnetic resonance spectroscopy (Echo MRI-4in 1/500™; Echo Medical Systems, Houston, TX, USA), before the diet intervention (Day 0) and the day of the necropsy (Day 21). For determination of the fecal lipid excretion, animals were placed in metabolic cages for 48 h starting at day 17. Apparent lipid absorption was calculated using lipid intake and excretion data after feces were collected for 48 h and the food intake was monitored over this time period.

Blood was collected into heparinized tubes from the caudal vein after 6 h of food restriction at days 3, 7 and 14. The day of the necropsy (Day 21), animals were anesthetized with isoflurane. Blood was collected from the abdominal aorta, and then RBC and plasma were separated by centrifugation at 626× *g* for 2 min. Plasma and RBC were stored at −80 °C until lipid analyses were carried out. Liver, brain, retina and spleen were removed and transferred to different vials, flash frozen with liquid nitrogen and stored at −80 °C until further analyses.

### 2.3. Lipid Extraction

Lipid extraction from organs (liver, spleen and brain) was described in details elsewhere [8]. Briefly, frozen tissues were pulverized, placed in chloroform, methanol, and water (2:1:0.8), homogenized and followed by a final Bligh and Dyer step to finish the lipid extraction. Total lipids were used to prepare fatty acid methyl esters (FAME) as described in the next section. A combination of methods was used to extract total lipids from feces [8]. Briefly, 1 g of dry frozen feces was macerated and lipids were extracted by the Bligh and Dyer method while heated at 60 °C to ensure complete extraction of total lipids. Lipids weight was obtained gravimetrically for fat absorption determination.

### 2.4. Fatty Acid Methyl Esters Preparation and Analysis by Gas Chromatography

FAMEs in plasma (60 μL) were prepared by mixing sample with a methanolic solution of hydrochloric acid (2 mL, 1.5 N, Supelco, Bellefonte, Palo Alto, CA, USA), methanol (2 mL) and *n*-hexane (1 mL) in a test tube. Tubes were then heated at 100 °C for 60 min [10]. After cooling down to room temperature, water (2 mL) was added and tubes were centrifuged at 2000 rpm for 4 min. The organic phase was collected for GC analyses. Direct methylation of RBC (100 μL) was performed as described for plasma having RBC previously washed with PBS buffer. Tissue fat was methylated from the fat extracted, as described in the previous section.

Analysis of total FAMEs was performed on a 7890 Agilent gas chromatograph (Agilent Technologies, Palo Alto, CA, USA), equipped with a fused-silica BPX-70 capillary column (10 m × 0.1 mm I.D., 0.2 μm film thickness; SGE, Melbourne, Australia). Split injector (25:1) and flame ionization detection (FID) systems were operating at 250 °C. Oven temperature programming was 50 °C isothermal for 0.2 min, increased to 180 °C at 120 °C/min, isothermal for 1 min at this temperature then increased to 220 °C at 20 °C/min and then to 250 °C at 50 °C/min. The carrier gas (H_2_) was maintained at a constant 1 mL/min and the acquisition of the FID signal at 100 Hz [13].

### 2.5. Statistical Analyses

The main comparisons assessed are the comparisons between the positive control FO + O and the treatment groups Vanil + O, Acetyl + O, and MAG + O. In order to ensure that the addition of Orlistat lowered the absorption of fatty acids, the positive control was also compared to the negative control in this case FO.

Data is presented as means ± standard error of the mean (S.E.M.), except for FA relative percentage in RBC, plasma, liver, spleen, retina and brain where median ± robust SEM are provided. For food intake, fat intake, fecal lipid excretion and apparent lipid absorption (%), an ANOVA and two-sided appropriate contrasts were calculated. For body weight parameters, for days 7, 14 and 21 on body weight, mixed models were performed with body weight at day 3 as covariate, day as continuous variable and subject as random variable. One-sided tests were calculated. For fat mass and lean mass parameters, an ANOVA with parameters at D-3 as covariate was performed. Appropriate one sided contrasts were calculated. For FA in different organs, Kruskal Wallis and exact Wilcoxon tests were performed. One-sided tests were calculated. Statistical calculations were performed using SAS software, version 9.1. Differences were considered as significant at *P* < 0.05.

## 3. Results

### 3.1. Body Weight, Food Intake and Apparent Lipid Absorption

Weight parameters, dietary intake, lipid absorption and calculated intake of EPA and DHA results for rats fed sample diets are shown in Table 2. Body weight increased for all groups with no significant differences observed. When groups were compared with changes obtained in group FO + O, no significant differences were observed except for body weight of the Acetyl + O group which was higher through time although not significantly different at day 20 (*P* = 0.0163, Table 2). 

Significantly higher lipid and daily food intake were observed in the group receiving Vanil + O, compared to the FO + O group. All the groups fed with Orlistat had significantly higher lipid excretion levels and reduced apparent lipid absorption relative to the FO receiving no Orlistat. Comparison of the data obtained in rats fed FO or FO + O diets demonstrates the efficiency of Orlistat to lower lipid absorption (−44.8% intake reduction), and therefore the consistency of the experimental approach. None of the MAG diets modified the action of Orlistat. Lipid absorption was reduced in all groups, compared to the control group receiving fish oil in absence of Orlistat. No significant differences were found for EPA intake between groups, while higher levels of DHA were found for the Acetyl + O and Vanil + O groups.

### 3.2. Incorporation of EPA in RBC, Plasma and Tissues

**Figure 2:**
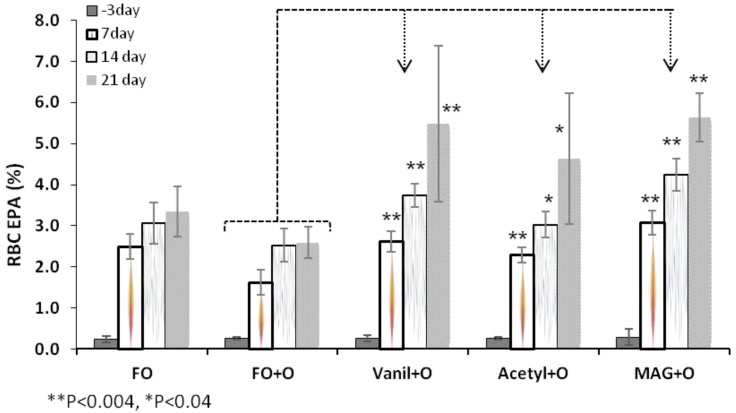
EPA in RBC at different time points, Median ±S.E.M.

At baseline, the EPA level in RBC was 0.2% of total FA and no differences were found among groups. The level of EPA in the groups fed Vanil + O, Acetyl + O and MAG + O were significantly higher at Day 21 when compared with groups fed control diet (FO + O), as shown in Table 3. Incorporation of EPA in RBC at different time points is shown in Figure 2 for all treatments. For rats fed FO + O, EPA level in RBC was significantly lower when compared with levels of EPA of rats fed FO diet with no Orlistat. These data are consistent with results obtained on lipid absorption and validate the fact that Orlistat, taken in conjunction with a dietary source of EPA (fish oil), lowers the incorporation of EPA in RBC.

**Table 3:** Level of EPA (g/100 g fatty acids) in RBC, plasma, liver, spleen, retina and brain in rats.

			Experimental Groups			
		FO	FO + O	Vanil + O	Acetyl + O	MAG + O
		Mean ± SD	Mean ± SD	Mean ± SD	Mean ± SD	Mean ± SD
RBC	Day 3	0.2 ± 0.1	0.3 ± 0.0	0.3 ± 0.1	0.3 ± 0.0	0.3 ± 0.2
	Day 21	3.3 ± 0.6 *	2.6 ± 0.4	5.5 ± 1.9 *	4.6 ± 1.6 **	5.6 ± 0.6 **
Plasma	Day 3	0.5 ± 0.1	0.6 ± 0.1	0.6 ± 0.1	0.7 ± 0.1	0.6 ± 0.1
	Day 21	4.5 ± 0.9	4.3 ± 0.9	8.5 ± 1.1 **	7.0 ± 1.5 **	8.5 ± 1.6 **
Liver		3.7 ± 0.74	3.1 ± 0.92	6.5 ± 1.57 **	5.2 ± 1.72 **	7.9 ± 2.14 **
Spleen		1.9 ± 0.57 *	1.1 ± 0.36	2.1 ± 0.8 *	2.1 ± 0.62 *	2.6 ± 0.59 **
Retina		0.3 ± 0.06 *	0.2 ± 0.05	0.4 ± 0.03 **	0.4 ± 0.08 **	0.45 ± 0.04 **
Brain		0.05 ± 0.01 **	0.1 ± 0.03	0.2 ± 0.02 **	0.1 ± 0.05	0.2 ± 0.03 **

*, **, *** indicate *P* < 0.05, 0.01 and 0.001, respectively; FO + O = Fish oil + Orlistat, Vanil + O = MAG Vanillin Acetal + Orlistat, Acetyl + O = Diacetylated MAG + Orlistat, MAG + O = MAG + Orlistat; Values are medians ± robust SD (*n* = 6). For statistics, data for groups fed fish oil diet or diets supplemented with MAG and MAG derivatives + Orlistat have been compared to the group receiving fish oil + Orlistat.

MAG + O diet showed the highest incorporation of EPA in RBC throughout the study, when compared to FO + O, followed by the Vanil + O and Acetyl + O groups as seen in Figure 2. These results show that MAG Vanillin Acetal, Diacetylated MAG and MAG are suitable carriers to efficiently deliver EPA to RBC compared with fish oil, even if Orlistat is present in the diets (Table 3, Figure 2). When Orlistat was not directly present in fish oil diets, evaluation of the level of EPA in groups fed with the different MAG diets, indicates that free MAG and MAG derivatives are efficient carriers of EPA when Orlistat is given orally. Indeed, the level of EPA was significantly higher in RBC of animals fed with MAG + O diet through time, than in the FO group and similar differences were observed for animals fed with the Vanil + O diet. 

**Figure 3:**
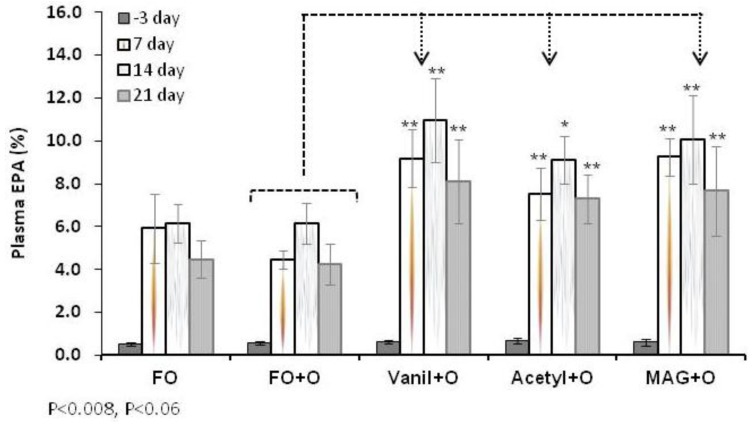
EPA in plasma at different time points, Median ± S.E.M.

In plasma, the EPA level at baseline was not significantly different among groups (Table 3). Figure 3 shows differences between animals fed FO diet and those fed with the control (FO + O) diet. Supplementation of fish oil diet with Orlistat tends to reduce EPA, especially at day 7, although this change was not significantly lower. For animals fed with MAG + O, Vanil + O and the Acetyl + O diets, the level of EPA was significantly higher, compared to the control diet. These results clearly corroborate the data obtained on RBC and show the potential of MAG and MAG derivatives to deliver EPA when lipid digestive function is impaired. 

The level of EPA in plasma lipids found in animals fed with MAG and MAG derivatives + Orlistat diets compared to animals fed the FO + O diet demonstrate as previously observed in RBC that free MAG and MAG derivatives are superior to FO to supply LC-PUFAs in this condition. Overall, at the end of the experiment (Day 21) levels of incorporation of EPA in plasma were higher in all groups when compared with the control group. However, at the last sampling point (Day 21) a trend towards decreased EPA is observed, potentially a consequence of the faster turnover that the plasma has in comparison with the RBC. 

As shown in Table 3, EPA levels in liver, spleen, retina and brain were evaluated. The EPA level of incorporation in liver, the central organ for lipid metabolism, was significantly lower for FO and FO + O groups. For the Vanil + O, Acetyl + O and MAG + O diets, levels were significantly higher when compared with control diet, with the highest level of EPA incorporation observed for MAG + O. A similar pattern of FAs was found for lipids of spleen with lower levels of EPA accumulation than for liver. In retina and brain, dietary composition is reflected by the incorporation of EPA. When compared with FO + O treatment, retina showed significantly higher levels for Vanil + O, Acetyl + O and MAG + O groups, whereas brain showed significant increments with Vanil + O, and MAG + O but not with Acetyl + O. 

### 3.3. Incorporation of DHA in RBC, Plasma and Tissues

The diets were made such that the EPA content was similar in all groups (approximately 4.4 g/kg of diet) with DHA levels in the range of 2.9–7.4 g/kg diet. However, results on DHA show it as one of the major LC-PUFA identified. Incorporation of DHA in RBC through time is shown in Table 4 for all treatments. The Vanil + O treatment produced a significantly higher DHA level in RBC when compared with the control diet. The level of DHA in plasma lipids was significantly higher in animals fed the FO diet compared to those fed the FO + O diet. Similar results were observed for levels of EPA. DHA level was significantly higher in groups that received MAG or MAG derivatives in diets, compared to animals fed the control diet. 

In liver, levels of DHA were significantly higher for Vanil + O, Acetyl + O and MAG + O treatments when compared with the control treatment. The level of incorporation of DHA in spleen, was significantly higher for the Vanil + O and the MAG + O treatment, although a lower level, when compared with the FO + O treatment. DHA level of incorporation with Acetyl + O treatment was also higher, although not significant. In retina, similar levels of DHA were observed (Table 4). 

**Table 4:** Level of DHA (g/100 g fatty acids) in RBC, plasma, liver, spleen, retina and brain in rats.

		Experimental Groups				
		FO	FO + O	Vanil + O	Acetyl + O	MAG + O
		Mean ± SD	Mean ± SD	Mean ± SD	Mean ± SD	Mean ± SD
RBC	Day 3	3.1 ± 0.2	3.2 ± 0.2	3.2 ± 0.1	3.0 ± 0.3	3.2 ± 0.2
	Day 21	4.1 ± 0.8	4.0 ± 0.3	4.9 ± 0.5 **	4.2 ± 0.6	4.3 ± 0.3
Plasma	Day 3	2.5 ± 0.2	2.3 ± 0.3	2.5 ± 0.2	2.6 ± 0.2	2.9 ± 0.2
	Day 21	5.7 ± 0.3 **	4.6 ± 0.6	7.7 ± 0.6 **	5.6 ± 0.8 **	6.4 ± 1.0 **
Liver		8.09 ± 1.13	7.41 ± 1.39	11.7 ± 3.31 **	9.4 ± 0.58 **	10.6 ± 1.27 **
Spleen		2.1 ± 0.68	1.4 ± 0.73	2.9 ± 0.91 **	2.4 ± 0.5	2.3 ± 0.46 *
Retina		35.1 ± 0.17	34.7 ± 1.26	35.5 ± 2.44	35.2 ± 1.33	35.2 ± 2.42
Brain		14.1 ± 0.51	13.7 ± 0.22	14.5 ± 0.22 **	14.4 ± 0.64	14.6 ± 0.34 **

*, **, *** indicate *P* < 0.05, 0.01 and 0.001, respectively; FO + O = Fish oil + Orlistat, Vanil + O = MAG Vanillin Acetal + Orlistat, Acetyl + O = Diacetylated MAG + Orlistat, MAG + O = MAG + Orlistat; Values are medians ± robust SD (*n* = 6). For statistics, data for groups fed fish oil diet or diets supplemented with MAG and MAG derivatives + Orlistat have been compared to the group receiving fish oil + Orlistat.

## 4. Discussion

In the current study, we wanted to explore if protected or free MAG lipids containing EPA and DHA has an influence on absorption and accretion in tissues under lipid malabsorption conditions. We selected three different glyceride derivatives that would, hypothetically require minimum digestion before crossing the gut barrier, and thus have an improved bioavailability of EPA and DHA. The tested molecules were: structured (**A**) vanillin acetal of *sn*-*2* MAG and (**B**) diacetyl derivative of *sn*-2 MAG and (C) free MAG mixture of isomers. A fourth group of rats were fed FO as a source of dietary TAG. The diets were made so that the EPA content was comparable (approximately 4.4 g/kg of diet) while DHA levels varied (2.9–7.4 g/kg diet). As expected from our previous experience [10], blood and tissue levels of EPA and DHA were lower in the Orlistat-fed group that induced malabsorption. In contrast, all experimental molecules were able to restore circulating and tissue levels of EPA in malabsorbing rats. These results suggest that absorption of structured or free MAG does not depend on lipolytic activities impaired by Orlistat. In the case of MAG vanillin acetal, the cleavage of the acetal moiety may happen at acidic pH in the stomach, and the resulting MAG likely being absorbed as such by the enterocytes. However, it is probable that the released *sn*-2 MAG isomerize readily in the lumen to *sn*-1(3)-isomers before absorption. The acyl migration is due to the fact that primary esters are more stable than the secondary esters. Results with the tested diacetylated MAG suggest that the occurrence of the two acetyl groups does not modify the polarity of the *sn*-2 MAG, allowing an efficient uptake by the intestinal cells. These results observed with the two structured MAG derivatives show that these molecules are efficient vehicles of EPA in malabsorption conditions. However, we recognize the limitations observed during production of structured MAG. Ethyl esters are used for the preparation of protected MAG and reaction yield is never complete, leaving them behind as ethyl esters of FA. Indeed, substantial amounts of ethyl ester of FA were detected in our product upon analyses. A part of the ethyl ester fraction can be removed by distillation, but the resulting material is never 100% pure. Therefore, the difference of purity between protected and free MAG may explain part of the difference observed. The free MAG utilized in our study was characterized as having exclusively *sn*-1(3) isomers of EPA [12]. Free MAG showed better absorption efficiencies and accretion to tissues when compared to the protected MAG molecules. It was our assumption that since EPA is esterified to *sn*-1(3) positions; it will require action of lipolytic enzymes. Given the fact that Orlistat is not a complete inhibitor of pTGL (pancreatic triglyceride lipases), the available activity might have produced glycerol, thus allowing better micellarization of cleaved EPA for optimal uptake. 

Indeed, this is the first study of its kind, where protected molecules of MAG have been tried and, therefore, comparison to existing data is not possible. However, many other strategies have been tried in past to overcome lipid malabsorption. For example, medium-chain triacylglycerols have been used for treatment of impaired intestinal lipolysis, or as a source of energy in parenteral nutrition. However, it increases total cholesterol concentration in primary hypertriglyceridemic subjects [14]. Alternatively, enzymatic interesterified fish oil with medium-chain FA with decanoic acid (10:0) at *sn*-1/3 positions showed an improved lymphatic transport of *n*-3 FA at 24 h in malabsorbing rats [15]. Another approach involves changing the molecular structure of the lipids, when possible. Cyclic FA monomers (CFAM) from linseed oil, acylated in specific positions, were tested in lymphatic FA transport and lipoprotein profile in rats’ lymph [16]. When structured TAGs were fed in a rat study, the chain length of medium-chain FA located in the primary position did not affect the lymphatic transport of LCFA in the *sn*-2 position [17]. Evidence indicated that 16:0 at the *sn*-2 position is absorbed as 2-MAG, and conserved through the process of TAG reassembly in the enterocytes. In piglets, a formula containing synthesized TAG with about 32% of 16:0 in the *sn*-2 position showed an increase of the 16:0 in *sn*-2 position in plasma and chylomicrons TAG but a reduction of ARA and DHA in phospholipids [18].

## 5. Conclusion

In summary, free MAG molecules investigated in the current study may find application in subjects experiencing lipases insufficiency. Orlistat is medically used in populations desiring weight loss, as it creates 40% reduction of intestinal lipolytic activity. While it may limit absorption of calorie-rich fats, it may also lead to impaired bioactive and essential FA status. However, it remains to be seen if these MAG or MAG derivatives would show similar effects in humans undergoing Orlistat therapy. It is noteworthy, however, to mention that our results cannot be generalized, but may also find application in malabsorption conditions with other underlying causes with additional research. However, the monumental task of developing preclinical and clinical model to address specific causes of malabsorption remains a necessary preliminary step. In conclusion, we demonstrated that malabsorption due to enzyme insufficiency may lead to decreased circulating and tissue levels of EPA and such a deficiency can be reversed using MAG provided as *sn*-1(3)-MAG or protected *sn*-2-MAG.
